# Recent trends in maternal and child health inequalities in Latin America and the Caribbean: analysis of repeated national surveys

**DOI:** 10.1186/s12939-023-01932-4

**Published:** 2023-07-01

**Authors:** Oscar J. Mujica, Antonio Sanhueza, Liliana Carvajal-Velez, Luis Paulo Vidaletti, Janaína C. Costa, Aluísio J. D. Barros, Cesar G. Victora

**Affiliations:** 1 Pan American Health Organization (PAHO), 525 23Rd Street NW, Washington, DC 20037 USA; 2 United Nations International Children Emergency Fund (UNICEF), New York City, USA; 3 Department of Global Public Health, Karolinska Institutet, Stockholm, Sweden; 4 International Center for Equity in Health (ICEH), Federal University of Pelotas, Pelotas, Brazil

**Keywords:** Health equity, Health status disparities, Child health, Maternal health, Trends, Latin America and the Caribbean

## Abstract

**Background:**

Although most Latin American and the Caribbean (LAC) countries made important progress in maternal and child health indicators from the 1990s up to 2010, little is known about such progress in the last decade. This study aims at documenting progress for each country as a whole, and to assess how within-country socioeconomic inequalities are evolving over time.

**Methods:**

We identified LAC countries for which a national survey was available between 2011–2015 and a second comparable survey in 2018–2020. These included Argentina, Costa Rica, Cuba, the Dominican Republic, Guyana, Honduras, Peru, and Suriname. The 16 surveys included in the analysis collected nationally representative data on 221,989 women and 152,983 children using multistage sampling. Twelve health-related outcomes were studied, seven of which related to intervention coverage: the composite coverage index, demand for family planning satisfied with modern methods, antenatal care (four or more visits and eight or more visits), skilled attendant at birth, postnatal care for the mother and full immunization coverage. Five additional impact indicators were also investigated: stunting prevalence among under-five children, tobacco use by women, adolescent fertility rate, and under-five and neonatal mortality rates. For each of these indicators, average annual relative change rates were calculated between the baseline and endline national level estimates, and changes in socioeconomic inequalities over time were assessed using the slope index of inequality.

**Results:**

Progress over time and the magnitude of inequalities varied according to country and indicator. For countries and indicators where baseline levels were high, as Argentina, Costa Rica and Cuba, progress was slow and inequalities small for most indicators. Countries that still have room for improvements, such as Guyana, Honduras, Peru and Suriname, showed faster progress for some but not all indicators, although also had wider inequalities. Among the countries studied, Peru was the top performer in terms of increasing coverage and reducing inequalities over time, followed by Honduras. Declines in family planning and immunization coverage were observed in some countries, and the widest inequalities were present for adolescent fertility and antenatal care coverage with eight or more visits.

**Conclusions:**

Although LAC countries are well placed in terms of current levels of health indicators compared to most low- and middle-income countries, important inequalities remain, and reversals are being observed in some areas. More targeted efforts and actions are needed in order to leave no one behind. Monitoring progress with an equity lens is essential, but this will require further investment in conducting surveys routinely.

**Supplementary Information:**

The online version contains supplementary material available at 10.1186/s12939-023-01932-4.

## Background

The 2030 Agenda for Sustainable Development includes 17 Goals (SDGs) [1], of which the third, or SDG3, consists of “ensuring healthy lives and promoting well-being for all at all ages” [2]. The wording of this goal, in particular the mention of “for all at all ages” echoes the overarching SDGs motto of “leaving no one behind” [3]. Consistently with the social determinants of health framework, the SDGs recognize that the pace of improvement will depend not only on changes in health services but also on broader societal changes to address inequalities related to wealth, education, residence, ethnicity and other drivers. The recent publication of the report of the Commission of the Pan American Health Organization on Equity and Health Inequalities in the Americas has highlighted the importance of social determinants in driving levels and distribution of health in the region and provided specific recommendations for multisectoral actions aimed at improving health status [4]. In consonance with the report, the Every Woman Every Child initiative for Latin America and the Caribbean (EWEC-LAC) has been providing further resources and technical guidance for countries to measure health equity and implement a multisectoral approach to women’s, children’s and adolescents’ health [5].

Although several Latin America and Caribbean (LAC) countries made important progress towards the achievement of the 2015 Millennium Development Goals (MDGs) [6], progress towards the SDGs is scarcely documented, as these goals were only defined in 2015. A special challenge faced by LAC is that it remains as one of the world regions with the highest levels of socioeconomic inequality [6], with large proportion of the population facing poverty and poor health outcomes [7–9].

The MDGs were rightfully criticized for measuring progress solely at the national level, without considering whether specific population subgroups – for example, the poor, rural residents or ethnic minorities – were being left behind [10]. This concern was addressed by SDG 17.18, which claims for disaggregated analyses of national statistics in order to monitor inequalities related to wealth, education, ethnicity and other relevant dimensions. Disaggregation is particularly important because inequalities affect access to health services and health outcomes among women, adolescents, and children [11, 12]. Accordingly, the EWEC-LAC initiative defined a set of core indicators to monitor health inequalities in the region [13] and the Pan American Health Organization has pushed the boundaries of the SDGs by proposing equity-specific targets [14]. Regular and frequent monitoring of inequalities is even more relevant in the context of the COVID-19 pandemic that may lead to the reversal of recent progress [15–18].

Two recent publications provided information on the baseline levels of SDG3-related inequalities in the LAC region by summarizing results on women, adolescents, and children from 21 countries with national surveys from 2011–2016 [19, 20]. The objective of the present analyses is to assess changes in the reported baseline levels, relying on data from recent surveys made available in 2018 or later for eight countries in the LAC region.

## Methods

### Data sources

Data were obtained from Demographic and Health Surveys (DHS) and Multiple Indicator Cluster Surveys (MICS). These two survey programs are highly comparable as they share similar sampling approaches and questionnaires designed to estimate standard health indicators [21, 22]. For Peru, data were obtained from the Peruvian Demographic and Family Health Surveys (ENDES) carried out annually by the DHS program up to 2015, and by the National Institute of Statistics thereafter keeping the same sampling methodology and questions [23]. To ensure comparability across surveys, all indicators were recalculated from the original microdata using a standard code that was written based on WHO/UNICEF definitions. All surveys used nationally representative samples obtained through multi-stage cluster sampling, with weights calculated according to the sampling probabilities of each cluster and individual, plus adjustments for non-response. Information was obtained on women aged 15–49 years and on children aged less than five years [22].

The database at the International Center for Equity in Health (www.equidade.org) includes over 450 surveys from more than 120 countries. We selected for analyses all countries from the LAC region with at least one survey carried out in 2018 or later (henceforth the endline survey) and an earlier survey carried out from 2010 to 2014 (the baseline survey).

### Health indicators

Seven health intervention coverage indicators were studied: the composite coverage index (CCI), demand for family planning satisfied with modern methods (mDFPS), antenatal care with four or more visits (ANC4) and with eight or more visits (ANC8), skilled attendant at birth (SAB), postnatal care for the mother (PNM) and full immunization coverage (FIC). Additionally, five impact indicators were studied: stunting prevalence among under-five children, tobacco use by women, adolescent fertility rate (AFR), under-five mortality rate (U5MR), and neonatal mortality rate (NMR). The CCI is an average of coverage with eight interventions along the continuum of care weighted in a way that gives the same weights to the four stages of the continuum represented in the indicator [24]. (All definitions for the indicators studied are presented in Supplementary Table S1). These indicators are both SDG3 and core EWEC-LAC indicators [25], except for ANC8 that has been proposed by the World Health Organization [26].

Not all indicators were available in all surveys. Specifically, the 2011 MICS from Argentina lacked the data required for several indicators; in addition, both surveys from Argentina are restricted to urban areas, which include more than 90% of the country’s population (https://www.statista.com/statistics/455778/urbanization-in-argentina/). The 2010 MICS from Cuba could not be included in the analyses due to lack of data on most indicators and on socioeconomic position; therefore the 2014 MICS was used as the baseline, although also missing information on asset scores; hence, wealth-based inequalities could not be estimated. Peru had several surveys since 2010, but information on postnatal care was only available from 2013 to 2019, because the 2020 results could not be used due to a high proportion of missing data. Argentina, Costa Rica, and Cuba did not collect information on fertility or mortality and were not included in these analyses.

### Inequality measures

Socioeconomic inequalities were assessed using household wealth indices based on assets and characteristics of the homes [27]. Households in each sample were classified according to wealth quintiles [Q1 (poorest 20%), Q2, Q3, Q4, Q5 (wealthiest 20%)]. As not all indicators or stratifiers are available for all countries missing information is noted in footnotes to tables and figures.

The slope index of inequality (SII), a summary measure of socioeconomic inequality, was calculated for each indicator in the baseline and endline surveys. It is calculated through logistic regression for coverage indicators and linear regression for mortality or fertility rates, and represents the absolute difference between the fitted values of the health indicator for the top and the bottom of the wealth distribution [28]. A SII of zero indicates no inequality, positive values indicate higher levels in the advantaged subgroups, or pro-rich inequality, and negative values indicate higher levels in the disadvantaged subgroups, or pro-poor inequalities. The SII is typically positive, or pro-rich, for intervention coverage indicators and negative, or pro-poor, for adverse health outcomes such as mortality indicators. For coverage or prevalence, the SII is expressed in percent points (pp); for child mortality it is expressed as deaths per 1,000 live births, and for adolescent fertility in births per 1,000 women-years.

### Analyses

We present baseline and endline estimates for all indicators, with the corresponding values of the SII, as defined above; 95% confidence intervals (95%CI) are provided in the Supplementary materials. Changes over time were estimated through the average annual relative change (AARC), which was calculated from the estimates (and their standard errors) for the first (i.e., baseline) and last (i.e., endline) surveys using variance weighted least squares regression of log-transformed estimates. The non-linear estimation function $\left(\left(1-{e}^{\beta }\right)\times -1\right)\times 100$ using Stata *nlcom* command was used to obtain the point and interval estimates of the AARC. It should be noted that the AARC provides the annual rate of change between the baseline and endline estimates, therefore efficiently controlling for the time elapsed between surveys. We also present two sets of equiplots that show the coverage or prevalence by wealth quintiles, allowing for a visual assessment of inequalities in the endline survey for each country.

All estimates were calculated from the original microdata for each survey according to standardized EWEC-LAC indicator definitions [13]. Analyses were carried out with Stata (StataCorp. 2019. Stata Statistical Software: Release 16. College Station, TX: StataCorp LLC), considering the sample design of each survey (clusters, weights, and strata).

## Results

Eight LAC countries had a national survey carried out in 2018 or later and an earlier survey between 2010 and 2014 (Table 1). In total, 221,989 women and 152,983 children were sampled in the 16 surveys used for the analysis. The 8 countries studied account for aproximately 20% of all women aged 15 to 49 years and 19% of all children aged less than 5 years in the LAC region by 2020. The Human Development Index (HDI) in 2020 ranged from 0.621 in Honduras to 0.840 in Argentina; on average, HDI was 0.756 among the eight countries (0.755 for LAC region). Compared to the recent SDG baseline medians from 21 LAC countries [19], the 8 countries studied had statistically significant lower levels for SAB (*P* < 0.0001) and higher levels for NMR (*P* = 0.0282), and U5MR (*P* = 0.0428); likewise, they had lower SII for mDFPS (*P* = 0.0019), and higher SII for ANC4 (*P* = 0.0002), SAB and PNM (*P* =  < 0.0001), and AFR (*P* = 0.0434). Therefore, the 8 countries studied tended to show worse health indicator and wider inequalities than the rest of the region.

**Table 1:** Countries included in the analyses, showing survey types and years

ISO code	Country	Year and survey type	Unweighted sample size	
			Women	Children
ARG	Argentina	2011 MICS	21,660	8,800
		2019 MICS	12,202	6,157
CRI	Costa Rica	2011 MICS	5,084	2,274
		2018 MICS	7,502	3,613
CUB	Cuba	2014 MICS	8,995	5,667
		2019 MICS	8,849	5,254
DOM	Dominican Republic	2013 DHS	9,372	3,714
		2019 MICS	22,295	8,422
GUY	Guyana	2014 MICS	5,076	3,358
		2019 MICS	5,887	2,801
HND	Honduras	2011 DHS	22,757	10,888
		2019 MICS	19,279	8,466
PER	Peru	2010 DHS	22,947	9,281
		2020 ENDES	35,430	62,222
SUR	Suriname	2010 MICS	6,290	3,308
		2018 MICS	6,999	4,234

*MICS* Multiple Indicator Cluster Survey, *DHS* Demographic and Health Survey, *ENDES* Encuesta Nacional de Demografía y Salud

**Fig. 1:**
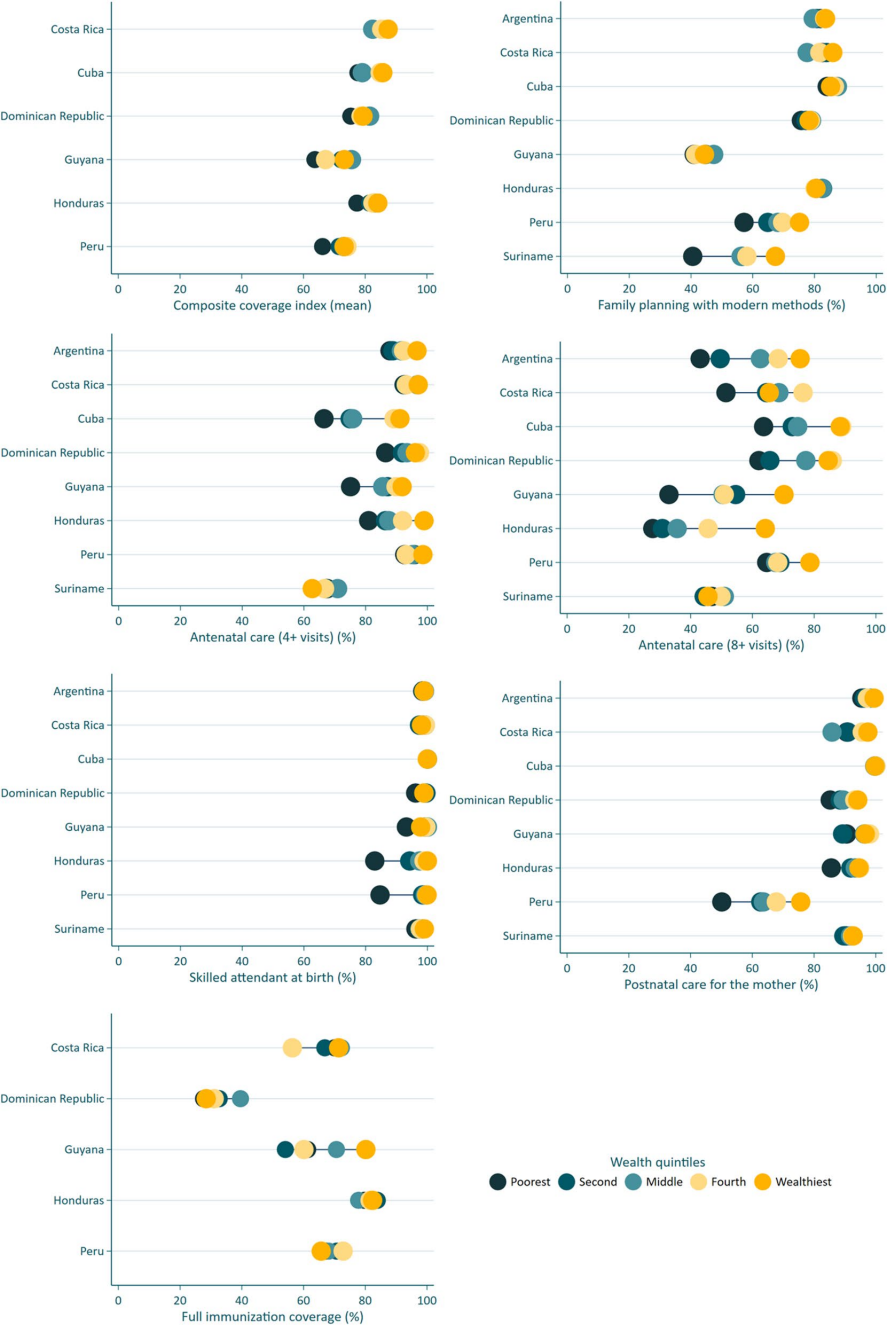
Coverage indicators according to wealth quintiles at the endline survey in each country

Table 2 shows the values of the seven coverage indicators and their corresponding SIIs in the baseline and endline survey, as well as the AARC for coverage and its 95% confidence intervals throughout the period of the study. When describing the results, we focus on countries for which the 95% intervals of the baseline and endline levels did not overlap, which are highlighted in green color when the change was significantly positive (increase in coverage or reduction in inequality) and in salmon color when the change was significantly negative (reduction in coverage or increase in inequality). Figure 1 shows coverage levels by wealth quintile (known as “equiplots”), in the most recent survey in each country. Corresponding *P* values are provided in Supplementary Table 2.

**Table 2:**
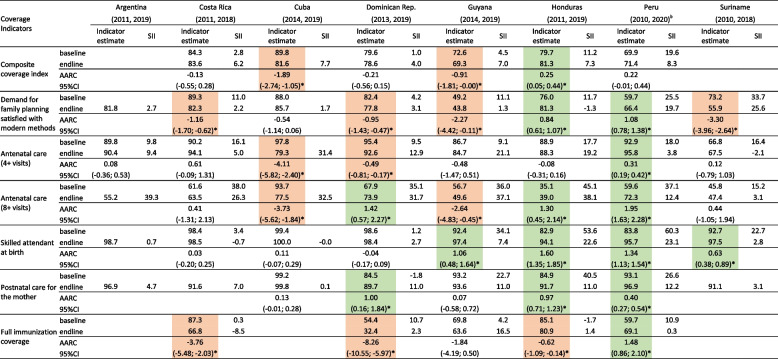
Coverage levels and corresponding slope indices of inequality (SII) at the baseline and endline surveys, and average relative annual change in coverage levels over the period^a^

^a^Results highlighted in green show significant (*) improvement over time (increase in coverage or reduction in inequality) and results highlighted in salmon show significant worsening over time (reduction in coverage or increase in inequality)

^b^Postnatal care in Peru refers to 2013 and 2019

### Composite coverage index

CCI coverage results were not available for Argentina and Suriname. Endline levels ranged from around 70% in Guyana and Peru to above 80% in Costa Rica and Cuba. Endline SII estimates were significantly positive in all countries except for the Dominican Republic and Guyana (Fig. 1 and Supplementary Table 2). The AARC for coverage were close to zero in most countries; the only significant change over time was the annual reduction of 1.8% in Cuba (*P* < 0.001). There were also no consistent time trends in the SII, except for a marked reduction from 19.3 to 8.6 percent points (pp) in Peru (*P* < 0.0001).

Demand for family planning satisfied with modern methods. Information was available for all countries, but Argentina lacked baseline information and trends could not be calculated. Endline coverage ranged from 43.8% in Guyana to 85.7% in Cuba. SII values were low and non-significant in six countries, but in Peru (SII: 19.7 pp) and Suriname (SII: 25.6 pp) important pro-rich inequalities were observed (Fig. 1 and Supplementary Table 2). Significant reductions in coverage over time were observed in Costa Rica and Dominican Republic (of around -1% per year) and Suriname (of around -3% per year), whereas there were significant increases in Honduras and Peru (of around + 1% per year). Honduras also showed a significant reduction in the SII, from 11.7 pp in 2011 to -1.3 pp in 2019 (*P* < 0.0001).

### Antenatal care

Information on the two ANC indicators was available for all countries. As expected, endline coverage for ANC4 (between 67.5% in Suriname and 95.8% in Peru) was higher than for ANC8 (from 39.0% in Honduras to 77.5% in Cuba). Inequalities at endline were also much more marked for ANC8 than for ANC4 (Fig. 1), to a maximum SII of 39.3 pp in Argentina. Increasing coverage levels over time, expressed by positive AARC for ANC8, were significant in the Dominican Republic, Honduras and Peru. The exception was Cuba with a significant reduction over time, which was driven by the high baseline coverage in 2014 of 93.7% that declined markedly to 77.5% in 2019 (P < 0.0001). Peru was the only country with a marked decline in the SII for ANC8, from 37.1 pp in 2010 to 12.4 pp in 2020 (*P* < 0.0001).

### Skilled attendant at birth

Information was available for all countries, with endline coverage ranging from 94.1% in Honduras to 100% in Cuba. Figure 1 shows that pro-rich inequality was very marked in Peru (23.1 pp) and Honduras (22.6 pp) but not in other countries given that baseline coverage was almost universal. For this reason, annual increases were small, but still reached significance in Guyana (1.06% per year), Honduras (1.60% per year), Peru (1.34% per year) and Suriname (0.63% per year). These four countries also showed very significant reductions in the SII over time (P < 0.0001), driven by a very high baseline inequality: from 34.1 to 7.4 pp in Guyana; from 53.6 to 22.6 pp in Honduras; from 60.3 to 23.1 pp in Peru; and from 22.7 to 2.8 pp in Suriname.

### Postnatal care for the mother

Information was available for all countries and endline coverage levels were 90% or higher. Pro-rich inequalities at endline were significant in all countries except for Costa Rica, Cuba and Suriname. Changes over time could not be calculated for Argentina, Costa Rica and Suriname because this indicator was not measured at baseline. Significant coverage increases over time were observed in Dominican Republic (*P* = 0.02), Honduras (*P* < 0.0001) and Peru (*P* < 0.0001), with the latter two countries also showing significant reductions in inequality (SII from 40.5 in 2011 to 11.1 pp in 2019; and from 26.6 in 2010 to 12.2 pp in 2020, respectively; *P* < 0.0001).

### Full immunization coverage

Results were not available for Argentina, Cuba and Suriname due to lack of information on one or more types of vaccines. Endline coverage ranged from 32.4% in the Dominican Republic to 80.9% in Honduras. Over time, coverage showed significant declines in Costa Rica (-3.76% per year; *P* < 0.0001) and the Dominican Republic (-8.26% per year; *P* < 0.0001), and an increase in Peru (+ 1.48% per year; *P* < 0.0001). No country showed significant reductions in inequality.

**Fig. 2:**
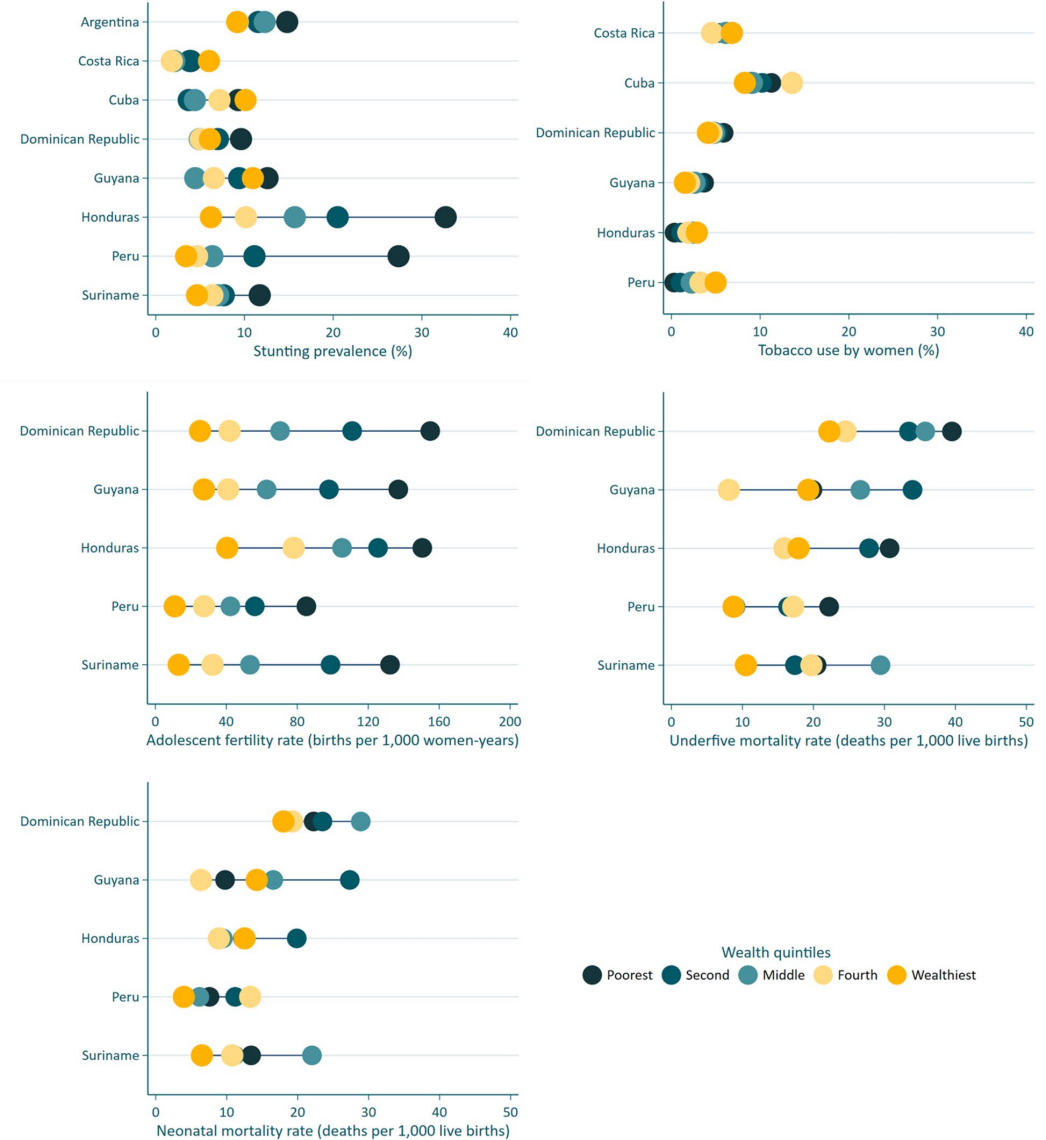
Prevalence of stunting, tobacco use by women, adolescent fertility rate, under-five and neonatal mortality rates according to wealth quintiles at the endline survey in each country

Table 3, Fig. 2 and Supplementary Table 3 show the results for the five impact outcomes other than the coverage indicators described above.

**Table 3:**
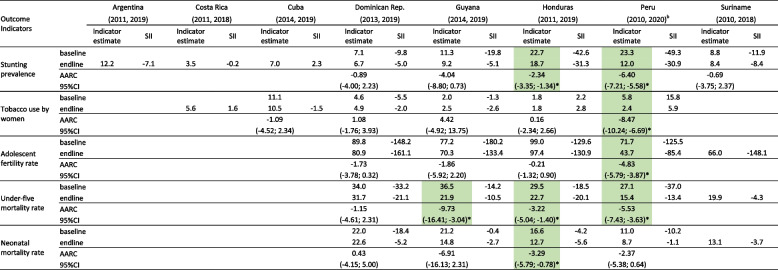
Levels and corresponding slope indices of inequality (SII) at the baseline and endline surveys for stunting, smoking, fertility and mortality indicators, and average relative annual change in coverage levels over the period^a^

^a^Results highlighted in green show significant (*) improvement over time (reduction in prevalence/rate or reduction in inequality)

### Stunting prevalence

Information was available for all countries. Endline levels ranged from 3.5% in Costa Rica to 18.7% in Honduras. Inequality in the endline survey (Fig. 2) was most marked in Peru and Honduras, with SII levels of -30.9 and -31.3 pp respectively. Inequalities were small in Costa Rica and Cuba where prevalence was low. AARC could be calculated for five countries, of which Peru and Honduras showed significant declines (-6.40% and – 2.34% per year, respectively). There was also an important reduction in inequality over time in these two countries: the SII fell from -49.3 in 2010 to -30.9 pp in 2020 in Peru (*P* < 0.0001), and from -42.6 in 2011 to -31.3 in 2019 in Honduras (*P* < 0.0001).

### Tobacco use by women

Information on this risk factor was not available for Argentina and Suriname. The highest prevalence in the most recent survey was found in Cuba (10.5%) and the lowest in Guyana, Honduras and Peru, all with 2.5% or less. In Honduras and Peru, wealthier women were more likely to smoke than poor women at endline, as shown by the significant positive values of the SII, but the reverse was observed in the Dominican Republic and Guyana where poor women were more likely to smoke (Supplementary Table 3). Peru was the only country to show a significant decline in smoking prevalence over time, at an average rate of -8.47% per year (Table 3). This was also accompanied by a decline in inequality, as smoking was 15.8 pp more prevalent among wealthier than among poor women at baseline, and this difference fell to 5.9 pp at endline (*P* < 0.0001).

The last three indicators refer to fertility and mortality, and information was not available from Argentina, Costa Rica and Cuba. In addition, Suriname only had information for the endline survey.

### Adolescent fertility rate

Honduras showed the highest endline rate of the five countries, with 97.4 annual births per 1,000 adolescents, whereas the lowest was observed in Peru, with 43.7. All SIIs were strongly and significantly negative (Fig. 2 and Supplementary Table 3), showing higher frequency among adolescents from poor families. Declining trends were observed in the four countries with data, but only reached statistical significance in Peru (*P* < 0.0001). Of the countries with data, only Peru showed significant changes over time in inequality: the SII went down from -125.5 in 2010 to -85.4 per 1,000 adolescents in 2020 (*P* = 0.02).

### Underfive mortality rate

Among the five countries with data, endline U5MR ranged from 15.4 deaths per 1,000 live births in Peru to 31.7 in the Dominican Republic. The five countries showed negative SIIs with higher mortality among poor children, but in Suriname and Guyana inequality was less marked and did not reach statistical significance. Of the four countries with time trend information, U5MR has dropped significantly in Guyana (-9.73% per year), Peru (-5.53% per year), and Honduras (-3.22% per year), but not in the Dominican Republic. Significant reduction in inequality was only observed in Peru, where the SII fell from -37.0 in 2010 to -13.4 per 1,000 live births in 2020 (*P* < 0.0001).

### Neonatal mortality rate

Results for NMR are similar to those for U5MR, with the highest endline rates in the Dominican Republic (22.6 per 1,000) and the lowest in Peru (8.7). Although all countries presented higher rates among the poorest children at endline, none of the five SII values were significant, nor were there any significant changes in inequality over time. Only Honduras showed a significant drop in NMR levels, from 16.6 in 2011 to 12.7 per 1,000 live births in 2019 (*P* = 0.01). Results for NMR must be interpreted with caution considering that it is a rare outcome, and therefore the statistical power for comparisons is lower than for any other outcome in the present analyses.

The results reported above for the SII are made evident when looking at the equiplots showing wealth-related inequalities in the endline survey (Figs. 1 and 2). As expected, inequalities are small when coverage gets closer to 100%. Guyana, Honduras and Peru showed the widest gaps, particularly the latter where a “bottom-inequality” pattern is observed for skilled birth attendance, with markedly lower coverage in the poorest than in all other quintiles. In contrast, inequalities in Argentina, Costa Rica, Cuba and the Dominican Republic were small. For Cuba, the exceptions were the two antenatal care indicators with important pro-rich inequalities. Suriname also showed narrow gaps, except for family planning.

When inequalities were evident in Figs. 1 and 2, pro-rich patterns in coverage prevailed, although in some cases the social gradients were not monotonic. For example, Costa Rica had an unusual pattern for full immunization coverage, with the lowest coverage observed in the fourth quintile; however, one needs to consider that this is the indicator with the smallest denominator (only children aged 12–23 months) and further breakdowns by wealth quintiles may result in lack of precision.

Wealth-based inequalities in child stunting and in tobacco smoking by women are shown graphically in Fig. 2. Except for stunting in Peru, with markedly higher prevalence in the poorest quintile, the magnitude of other inequalities is small.

Results for fertility and mortality were only available for five countries (Fig. 2), all of which show remarkably wide inequalities in adolescent fertility. Social gradients in under-five mortality –and to a lesser extent in neonatal mortality– are present in all countries. However, gradients are not always monotonic, which is possibly related to statistical imprecision given that mortality is a rare outcome in the region.

## Discussion

Compared to most other low- and middle-income countries in the world, those from the LAC region are characterized by relatively good performance in terms of women’s, adolescents’ and children’s health [29]. Our analyses of recent trends in eight countries, however, show that the performance of LAC countries is heterogeneous, and that important inequalities remain within several of them.

Three of the countries studied – Argentina, Costa Rica and Cuba – showed high baseline and endline coverage levels for most indicators and relatively narrow social inequalities with the exception of coverage with eight or more antenatal visits. For the two countries where it was possible to study time trends for most indicators, Cuba showed significant coverage reductions in CCI and in both antenatal care indicators, and Costa Rica in family planning and full immunization coverage. Data on the latter were not available for Cuba or Argentina. Results for these three countries must be interpreted in light of their relatively high baseline levels and therefore limited scope for further improvements, but the declines in coverage are noteworthy.

At the other extreme, four countries showed below-average baseline coverage and wider inequalities for most indicators: Guyana, Honduras, Peru and Suriname. For most indicators, these countries had substantial room for improvement. Of the eight countries included in our analyses, Peru was the top performer in terms of increasing coverage, reducing stunting and mortality, and narrowing down socioeconomic inequalities. With positive trends over time observed for 10 of the 12 indicators, Peru was also the only country to show improvements in full immunization (increased coverage) as well as decreases in tobacco use by women and in adolescent fertility. The second positive outlier was Honduras where there were significant improvements in levels for six of the 12 indicators. Progress in Suriname and Guyana was not so clearcut, and the progress in Peru had already been highlighted in the literature as associated to an antipoverty political agenda [30]. On the negative side, Peru remained as a country with wider socioeconomic inequalities at endline than most countries in our analyses, as summarized by the SII.

Finally, the Dominican Republic did not quite fit in either of the two groups of countries described above. It had the highest under-five mortality rate of the eight countries, which is consistent with international data sources [31], yet prevalence of stunting was the second lowest after Cuba. Coverage was reasonably high for most indicators, except for full immunization and antenatal care (eight or more visits). Socioeconomic inequalities were also rather small except for adolescent fertility and antenatal care. In terms of progress over time, there were noticeable reductions in family planning and immunization coverage, the latter having peaked in 2006 at 59% and declining to 32% by 2019 (data not shown).

We now turn to a discussion of the most remarkable findings for each indicator. The two coverage indicators with the worst performance over time were family planning (with significant declines in three out of six countries with data) and full immunization (with declines in two out of five countries) [32, 33]. Earlier analyses of family planning in the region pointed to the need to revigorate national programs, particularly the use of long-acting reversible methods [34].

On the positive side, skilled attendance at birth increased in four out of seven countries with data and antenatal care (8 + visits) in three out of seven. There was hardly any progress in terms of the CCI, for which the only significant yet adverse change was a decline in Cuba.

Regarding outcomes other than coverage, stunting prevalence fell significantly in two countries, tobacco smoking by women and adolescent fertility only fell in Peru, whereas under-five mortality fell in three out of five countries with data [35].

The most remarkable socioeconomic inequalities were observed for adolescent fertility with all five countries with data showing five-fold or higher gaps between the richest and poorest quintiles. Earlier publications had already highlighted similar findings throughout the LAC region [36]. In most countries, inequality in coverage with eight or more antenatal care visits also were wider than those for four or more visits. This is not surprising given that, according to the literature, more demanding indicators, requiring a larger number of contacts with health workers, tend to be more inequitable as poorer women are likely to have less access [37, 38]. It is important to recall that WHO recommends at least eight prenatal care visits to reduce perinatal mortality and improve women’s experience of care [26].

Our analyses have limitations. Data for the analyses were only available for eight of the 33 LAC countries that account for only 19.6% of the regional population, and only four countries had complete trend data on the selected indicators. Using these data, it is not possible to provide a comprehensive picture of regional trends, particularly when the three most populous countries in the region – Brazil, Mexico, and Colombia – could not be included due to lack of comparable data in the 2 periods of analysis. Even among the eight countries, surveys from Argentina, Cuba and Suriname lacked several indicators. Time trend analyses are also affected by different time periods between the endline and baseline surveys; countries with only two surveys that are close in time such as Cuba and Guyana may provide less reliable time trend estimates than countries with three or more surveys spread throughout the whole decade. Peru has had annual surveys from 2010 to 2020 and this may be one of the reasons why changes over time were so marked, even in spite of a pandemic year. Furthermore, any non-linear evolution in the average or the inequality of particular health indicators experienced by countries over time cannot be captured by an analysis based on two points in time, as presented in our study.

Other limitations are related to the indicators under analysis. Data on antenatal, delivery and postnatal care refer to births that took place during the five (DHS) or two (MICS) years prior to the survey, thus failing to pick up recent progress. Maternal recall of events that took place up to five years before the interview may also be affected by long recall periods. Lack of precision may result from small sample sizes in some surveys for rare events (such as neonatal mortality), particularly when stratified by wealth quintiles. For maternal schooling, some groups (such as no education or primary only) are small in countries where most women completed secondary education, as in Cuba.

The strengths of the analyses include reliance on nationally representative, population-based surveys with consistent methodology and use of standardized indicator definitions. Results were based on reanalysis of the raw data carried out by a single team, and different dimensions of inequality were used.

We recognize at least two other limitations in data availability in our study. Firstly, there is a lack of standardized data collected on the quality of care in the region, with some studies estimating that poor quality of care may account for over half of treatable condition-related deaths [39]. Inequalities in the quality of care received by people in different strata could be important drivers of poor health outcomes. Secondly, most health surveys in the region only collect data from women, which limits comparisons across genders, as well as analysis of the implications of gender and health. Analyzing gender disparities in health is still a significant challenge in the region [40, 41] and is especially important for addressing risk factors such as, for instance, tobacco use and physical activity. These two relevant dimensions of inequality cannot be ascertained in our study.

Our paper is about a multicountry study aimed at describing recent inequality trends. Explaining the findings described here, and understanding the underlying mechanisms and drivers of success (or lack thereof) deserves in-depth studies, with different and specific study designs and the use of quantitative and qualitative methods to explore both the overall political and socioeconomic context as well as the effectiveness and potential impact of specific pro-equity policies put in place. Such in-depth studies are outside the scope of our paper, but we hope that our findings will drive further studies on those important questions.

Our findings, and the limitations of our analyses, highlight the pressing need for more frequent collection of data using population-based surveys to meet the requirement of SDG 17.18 for disaggregated analyses. The situation is even more pressing giving the fact that survey activities were interrupted in many countries starting in 2020 and in 2021 due to the COVID-19 pandemic. A new round of surveys in all 35 countries in the region is urgently needed in order to document the impact of COVID-19 on service coverage, nutrition and mortality, and to provide essential information for planning the recovery efforts.

## Conclusions

Although LAC countries are well placed in terms of current levels of health indicators compared to most low- and middle-income countries, in those for which survey data for trend analysis is available important inequalities remain, and reversals are being observed in some areas. More targeted efforts and actions on the proximal and distal determinants of maternal and child health are needed in order to leave no one behind by 2030 and beyond. Monitoring progress with an equity lens is essential, but this will require further investment in conducting surveys routinely,including collecting standardized and comparable quality of care data, as well as in strengthening national and subnational health information systems in the region.

## Supplementary Information

**Additional file 1:** **Table S1.** Indicator definitions. **Table S2.** Coverage levels and corresponding slope indices of inequality (SII) at the baseline and endline surveys, showing 95% confidence intervals. **Table S3.** Levels and corresponding slope indices of inequality (SII) at the baseline and endline surveys for stunting, smoking, fertility and mortality indicators, showing 95% confidence intervals.

## Data Availability

This analysis used publicly available data collected by third parties. The data used in this study can be accessed directly through the DHS and MICS data repositories.

## Abbreviations

AARC: Average annual relative change
AFR: Adolescent fertility rate
ANC4: Antenatal care with four or more visits
ANC8: Antenatal care with eight or more visits
CCI: Composite coverage index
COVID-19: Coronavirus disease of 2019
DHS: Demographic and Health Surveys
ENDES: Encuesta Nacional de Demografía y Salud (Demographic and Family Health Survey)
EWEC-LAC: Every Woman Every Child Initiative for Latin America and the Caribbean
FIC: Full immunization coverage
ICEH: International Center for Equity in Health
LAC: Latin America and the Caribbean
mDFPS: Demand for family planning satisfied with modern methods
MDGs: Millennium Development Goals
MICS: Multiple Indicator Cluster Surveys
NMR: Neonatal mortality rate
PAHO: Pan American Health Organization
PNM: Postnatal care for the mother
pp: Percent points
SAB: Skilled attendant at birth
SDGs: Sustainable Development Goals
SII: Slope index of inequality
U5MR: Under-five mortality rate
UNICEF: United Nations International Children Emergency Fund
WHO: World Health Organization
