# Supplementary material for: Recent trends in maternal and child health inequalities in Latin America and the Caribbean: analysis of repeated national surveys
Source: Int J Equity Health. 2023 Jul 1;22:125. doi: 10.1186/s12939-023-01932-4 (PMC10314462; doi:10.1186/s12939-023-01932-4)
Supplement: Supplementary file 1 — Additional file 1: Table S1. Indicator definitions. Table S2. Coverage levels and corresponding slope indices of inequality (SII) at the baseline and endline surveys, showing 95% confidence intervals. Table S3. Levels and corresponding slope indices of inequality (SII) at the baseline and endline surveys for stunting, smoking, fertility and mortality indicators, showing 95% confidence intervals. [file 12939_2023_1932_MOESM1_ESM.docx]

**SUPPLEMENTARY INFORMATION**

**Additional File 1**

**Supplement to:**  Mujica OJ, Sanhueza A, Carvajal-Velez L, Vidaletti LP, Costa JC, Barros AJD, Victora CG. Recent trends in maternal and child health inequalities in Latin America and the Caribbean: analysis of repeated national surveys.

**Table of Content:**

| Table S1. | Indicator definitions ………………………………………………………………………………………………………………………………….. | 2 |
| --- | --- | --- |
| Table S2. | Coverage levels and corresponding slope indices of inequality (SII) at the baseline and endline surveys, showing 95% confidence intervals …………………………………………………………………………………………………………….. | 3 |
| Table S3. | Levels and corresponding slope indices of inequality (SII) at the baseline and endline surveys for stunting, smoking, fertility and mortality indicators, showing 95% confidence intervals ………………………………………….. | 4 |

**Table S1. Indicator definitions**

| **Indicator** | **Denominator** | **Numerator** |
| --- | --- | --- |
| **Composite coverage index (CCI)** | See below for the denominators of each component of the CCI | Weighted average of the coverage of eight interventions: (1) family planning coverage with modern methods; (2) skilled birth attendant; (3) at least four antenatal care visits by a skilled provider; (4) BCG vaccination; (5) three DTP vaccinations; (6) measles vaccination; (7) Oral rehydration salts therapy for diarrhea; and (8) care-seeking for childhood pneumonia. |
| **Demand for family planning satisfied with modern methods** | Women aged 15-49 years currently married or in a union in need of contraception | Who are using (or whose partner is using) any modern method as defined by PAHO/CLAP https://www.paho.org/es/centro-latinoamericano-perinatologia-salud-mujer-reproductiva-clap) |
| **Antenatal care (four or more visits)** | Women aged 15-49 years who had a birth in the last 3 years (last birth) | Women with at least four antenatal care visits with any provider |
| **Antenatal care (eight or more visits)** | Women aged 15-49 years who had a birth in the last 3 years (last birth) | Women with at least eight antenatal care visits with any provider |
| **Skilled attendant at delivery** | All live births in the last 3 years | Children delivered by a skilled attendant |
| **Postnatal care for mothers** | Women aged 15-49 years who gave birth in the last 2 years (last birth) | Women who received postnatal care within 2 days of delivery |
| **Full immunization coverage** | Live children aged 12-23 months (18-29 months in Peru due to a different schedule) | Children who received 3 doses of DPT & 3 doses of polio & 1 dose of measles & 1 dose of BCG |
| **Stunting in children** | Live children under 5 years, length/height measured | With height-for-age < -2SD |
| **Female tobacco use** | Women aged 15-49 years | Who used any type of tobacco in the last one month |
| **Adolescent fertility rate** | Number of women-years of exposure in the 1–60 months before the survey of women aged 15-19 years | Number of births that occurred in the 1–60 months before the survey to women aged 15-19 years at the time of the birth |
| **Under-five mortality rate** | Number of surviving children at beginning of specified age range during the specified time period | Deaths at ages 0 to 59 months, including deaths reported at age zero months |
| **Neonatal mortality rate** | Number of surviving children at beginning of specified age range during the specified time period | Deaths at ages 0 to 30 days, including |

**Table S2 Coverage levels and corresponding slope indices of inequality (SII) at the baseline and endline surveys, showing 95% confidence intervals.**

| Coverage  Indicators |  |  | Argentina | | Costa Rica | | Cuba | | Dominican Rep. | | Guyana | | Honduras | | Peru | | Suriname | |
| --- | --- | --- | --- | --- | --- | --- | --- | --- | --- | --- | --- | --- | --- | --- | --- | --- | --- | --- |
|  |  |  | (2011, 2019) | | (2011, 2018) | | (2014, 2019) | | (2013, 2019) | | (2014, 2019) | | (2011, 2019) | | (2010, 2020)^b^ | | (2010, 2018) | |
|  |  |  | Indicator estimate | SII | Indicator estimate | SII | Indicator estimate | SII | Indicator estimate | SII | Indicator estimate | SII | Indicator estimate | SII | Indicator estimate | SII | Indicator estimate | SII |
| Composite  coverage  index |  |  |  |  | p value = 0.53 | p value = 0.36 | p value < 0.001 |  | p value = 0.25 | p value = 0.42 | p value = 0.05 | p value = 0.70 | p value = 0.01 | p value = 0.11 | p value = 0.06 | p value < 0.001 |  |  |
|  | baseline | estimate |  |  | 84.3 | 2.8 | 89.8 |  | 79.6 | 1.0 | 72.6 | 4.5 | 79.7 | 11.2 | 69.9 | 19.6 |  |  |
|  |  | 95%CI |  |  | (82.4; 86.2) | (-4.2; 9.7) | (87.6; 92.1) |  | (78.3; 80.9) | (-4.9; 6.9) | (70.9; 74.2) | (-2.9; 11.9) | (78.9; 80.5) | (7.9; 14.5) | (68.8; 70.9) | (15.2; 24.1) |  |  |
|  | endline | estimate |  |  | 83.6 | 6.2 | 81.6 | 7.7 | 78.6 | 4.0 | 69.3 | 7.0 | 81.3 | 7.3 | 71.4 | 8.3 |  |  |
|  |  | 95%CI |  |  | (82.1; 85.1) | (3.8; 8.6) | (78.8; 84.5) | (1.4; 14.0) | (77.5; 79.7) | (-0.5; 8.4) | (66.6; 72.0) | (-3.4; 17.4) | (80.3; 82.3) | (3.9; 10.7) | (70.2; 72.6) | (2.2; 14.4) |  |  |
| Demand for family planning satisfied with modern methods |  |  |  |  | p value < 0.001 | p value = 0.10 | p value = 0.08 |  | p value < 0.001 | p value = 0.79 | p value = 0.04 | p value = 0.10 | p value < 0.001 | p value < 0.001 | p value < 0.001 | p value = 0.06 | p value < 0.001 | p value = 0.13 |
|  | baseline | estimate |  |  | 89.3 | 11.0 | 88.0 |  | 82.4 | 4.2 | 49.2 | 11.1 | 76.0 | 11.7 | 59.7 | 25.5 | 73.2 | 33.7 |
|  |  | 95%CI |  |  | (86.6; 91.4) | (2.8; 19.2) | (85.8; 89.9) |  | (80.4; 84.3) | (-2.4; 10.7) | (46.2; 52.1) | (2.8; 19.3) | (75.0; 77.0) | (8.1; 15.2) | (58.3; 61.0) | (21.1; 30.0) | (70.6; 75.6) | (26.5; 40.8) |
|  | endline | estimate | 81.8 | 2.7 | 82.3 | 2.2 | 85.7 | 1.7 | 77.8 | 3.1 | 43.8 | 1.3 | 81.3 | -1.3 | 66.4 | 19.7 | 55.9 | 25.6 |
|  |  | 95%CI | (79.9; 83.6) | (-3.9; 9.4) | (79.9; 84.4) | (-4.5; 8.9) | (83.9; 87.2) | (-4.8; 8.3) | (76.4; 79.2) | (-1.3; 7.5) | (39.8; 48.0) | (-7.1; 9.7) | (80.2; 82.3) | (-4.9; 2.3) | (65.2; 67.7) | (15.5; 23.9) | (53.5; 58.3) | (18.0; 33.2) |
| Antenatal care  (4+ visits) |  |  | p value = 0.72 | p value = 0.95 | p value = 0.08 | p value = 0.25 | p value < 0.001 |  | p value < 0.001 | p value = 0.27 | p value = 0.34 | p value = 0.12 | p value = 0.52 | p value = 0.59 | p value < 0.001 | p value < 0.001 | p value = 0.79 | p value = 0.02 |
|  | baseline | estimate | 89.8 | 9.8 | 90.2 | 16.1 | 97.8 |  | 95.4 | 9.5 | 86.7 | 9.1 | 88.9 | 17.7 | 92.9 | 18.0 | 66.8 | 16.4 |
|  |  | 95%CI | (87.5; 91.6) | (2.4; 17.2) | (85.6; 93.4) | (-0.6; 32.8) | (95.8; 98.8) |  | (94.0; 96.5) | (5.7; 13.2) | (84.2; 88.9) | (1.0; 17.1) | (88.0; 89.7) | (14.8; 20.6) | (91.9; 93.8) | (13.6; 22.5) | (63.4; 70.0) | (5.6; 27.3) |
|  | endline | estimate | 90.4 | 9.4 | 94.1 | 5.0 | 79.3 | 31.4 | 92.6 | 12.9 | 84.7 | 21.1 | 88.3 | 19.2 | 95.8 | 3.8 | 67.5 | -2.1 |
|  |  | 95%CI | (87.6; 92.6) | (1.5; 17.4) | (91.4; 96.0) | (-3.6; 13.7) | (71.3; 85.5) | (7.9; 54.9) | (91.2; 93.8) | (8.1; 17.7) | (80.8; 87.9) | (8.1; 34.1) | (86.8; 89.7) | (14.4; 24.0) | (95.3; 96.2) | (2.0; 5.5) | (63.7; 71.0) | (-14.1; 9.8) |
| Antenatal care  (8+ visits) |  |  |  |  | p value = 0.63 | p value = 0.39 | p value < 0.001 |  | p value < 0.001 | p value = 0.55 | p value = 0.02 | p value = 0.89 | p value < 0.001 | p value = 0.07 | p value < 0.001 | p value < 0.001 | p value = 0.56 | p value = 0.18 |
|  | baseline | estimate |  |  | 61.6 | 38.0 | 93.7 |  | 67.9 | 35.1 | 56.7 | 36.0 | 35.1 | 45.1 | 59.6 | 37.1 | 45.8 | 15.2 |
|  |  | 95%CI |  |  | (55.9; 67.1) | (21.2; 54.7) | (89.0; 96.5) |  | (65.1; 70.6) | (27.0; 43.2) | (53.2; 60.2) | (24.6; 47.4) | (33.7; 36.6) | (41.3; 49.0) | (57.9; 61.2) | (31.7; 42.5) | (42.1; 49.5) | (2.7; 27.6) |
|  | endline | estimate | 55.2 | 39.3 | 63.5 | 26.3 | 77.5 | 32.5 | 73.9 | 31.7 | 49.6 | 37.1 | 39.0 | 38.1 | 72.3 | 12.4 | 47.4 | 3.1 |
|  |  | 95%CI | (50.8; 59.5) | (27.4; 51.2) | (58.4; 68.3) | (5.3; 47.2) | (69.7; 83.7) | (9.4; 55.6) | (71.5; 76.1) | (24.5; 39.0) | (45.0; 54.3) | (24.8; 49.4) | (36.9; 41.0) | (31.6; 44.6) | (71.2; 73.4) | (8.8; 16.1) | (43.4; 51.5) | (-9.6; 15.7) |
| Skilled attendant  at birth |  |  |  |  | p value = 0.82 | p value = 0.25 | p value = 0.23 |  | p value = 0.56 | p value = 0.41 | p value < 0.001 | p value < 0.001 | p value < 0.001 | p value < 0.001 | p value < 0.001 | p value < 0.001 | p value < 0.001 | p value < 0.001 |
|  | baseline | estimate |  |  | 98.4 | 3.4 | 99.4 |  | 98.6 | 1.2 | 92.4 | 34.1 | 82.9 | 53.6 | 83.8 | 60.3 | 92.7 | 22.7 |
|  |  | 95%CI |  |  | (96.5; 99.2) | (-2.9; 9.6) | (97.2; 99.9) |  | (98.0; 99.0) | (-1.3; 3.7) | (89.6; 94.5) | (20.7; 47.5) | (81.5; 84.1) | (50.0; 57.3) | (82.2; 85.4) | (55.0; 65.6) | (90.9; 94.1) | (13.5; 31.8) |
|  | endline | estimate | 98.7 | 0.7 | 98.5 | -0.7 | 100.0 | -0.0 | 98.4 | 2.7 | 97.4 | 7.4 | 94.1 | 22.6 | 95.7 | 23.1 | 97.5 | 2.8 |
|  |  | 95%CI | (98.0; 99.2) | (-1.5; 2.9) | (97.2; 99.2) | (-3.6; 2.2) | (99.9; 100.0) | (-0.1; 0.0) | (97.7; 98.9) | (0.2; 5.1) | (96.1; 98.3) | (1.3; 13.6) | (92.9; 95.1) | (17.2; 28.0) | (95.1; 96.3) | (18.9; 27.3) | (96.3; 98.3) | (-0.5; 6.1) |
| Postnatal care  for the mother |  |  |  |  |  |  | p value = 0.07 |  | p value = 0.02 | p value = 0.03 | p value = 0.83 | p value = 0.06 | p value < 0.001 | p value < 0.001 | p value < 0.001 | p value < 0.001 |  |  |
|  | baseline | estimate |  |  |  |  | 99.2 |  | 84.5 | -1.8 | 93.2 | 22.7 | 84.9 | 40.5 | 93.1 | 26.6 |  |  |
|  |  | 95%CI |  |  |  |  | (98.1; 99.6) |  | (80.0; 88.1) | (-12.3; 8.7) | (90.9; 95.0) | (13.3; 32.2) | (83.5; 86.2) | (35.9; 45.2) | (91.9; 94.1) | (20.2; 33.1) |  |  |
|  | endline | estimate | 96.9 | 4.7 | 91.6 | 7.0 | 99.8 | 0.1 | 89.7 | 11.0 | 93.6 | 11.0 | 91.7 | 11.0 | 96.9 | 12.2 | 91.1 | 3.1 |
|  |  | 95%CI | (95.6; 97.9) | (0.7; 8.8) | (88.1; 94.1) | (-2.6; 16.7) | (99.6; 99.9) | (-0.4; 0.6) | (88.2; 91.0) | (6.2; 15.7) | (90.9; 95.5) | (3.3; 18.8) | (90.5; 92.9) | (6.4; 15.7) | (96.3; 97.4) | (8.5; 15.8) | (89.1; 92.7) | (-2.6; 8.9) |
| Full immunization coverage |  |  |  |  | p value < 0.001 | p value = 0.61 |  |  | p value < 0.001 | p value = 0.47 | p value = 0.13 | p value = 0.33 | p value = 0.01 | p value = 0.57 | p value < 0.001 | p value = 0.13 |  |  |
|  | baseline | estimate |  |  | 87.3 | 0.3 |  |  | 54.4 | 10.7 | 69.8 | 4.2 | 85.1 | -1.7 | 59.7 | 10.9 |  |  |
|  |  | 95%CI |  |  | (80.6; 91.9) | (-22.9; 23.6) |  |  | (48.3; 60.3) | (-9.5; 30.8) | (64.5; 74.7) | (-9.2; 17.6) | (82.7; 87.1) | (-8.9; 5.5) | (56.5; 62.8) | (-0.4; 22.2) |  |  |
|  | endline | estimate |  |  | 66.8 | -8.5 |  |  | 32.4 | 2.3 | 63.6 | 16.5 | 80.9 | 1.4 | 69.1 | 0.3 |  |  |
|  |  | 95%CI |  |  | (59.2; 73.6) | (-33.9; 16.8) |  |  | (29.2; 35.8) | (-8.3; 12.9) | (57.3; 69.5) | (-4.3; 37.3) | (78.6; 83.1) | (-6.5; 9.3) | (66.8; 71.2) | (-7.5; 8.1) |  |  |

Note: (a) Postnatal care in Peru refers to 2013 and 2019.

**Table S3. Levels and corresponding slope indices of inequality (SII) at the baseline and endline surveys for stunting, smoking, fertility and mortality indicators, showing 95% confidence intervals.**

| Outcome  Indicators |  |  | Argentina | | Costa Rica | | Cuba | | Dominican Rep. | | Guyana | | Honduras | | Peru | | Suriname | |
| --- | --- | --- | --- | --- | --- | --- | --- | --- | --- | --- | --- | --- | --- | --- | --- | --- | --- | --- |
|  |  |  | (2011, 2019) | | (2011, 2018) | | (2014, 2019) | | (2013, 2019) | | (2014, 2019) | | (2011, 2019) | | (2010, 2020)^b^ | | (2010, 2018) | |
|  |  |  | Indicator estimate | SII | Indicator estimate | SII | Indicator estimate | SII | Indicator estimate | SII | Indicator estimate | SII | Indicator estimate | SII | Indicator estimate | SII | Indicator estimate | SII |
| Stunting prevalence |  |  |  |  |  |  |  |  | p value = 0.58 | p value = 0.07 | p value = 0.10 | p value < 0.001 | p value < 0.001 | p value < 0.001 | p value < 0.001 | p value < 0.001 | p value = 0.65 | p value = 0.40 |
|  | baseline | estimate |  |  |  |  |  |  | 7.1 | -9.8 | 11.3 | -19.8 | 22.7 | -42.6 | 23.3 | -49.3 | 8.8 | -11.9 |
|  |  | 95%CI |  |  |  |  |  |  | (6.1; 8.2) | (-14.0; -5.7) | (9.6; 13.2) | (-26.5; -13.1) | (21.5; 23.9) | (-46.1; -39.0) | (22.0; 24.6) | (-53.0; -45.7) | (7.6; 10.2) | (-17.1; -6.6) |
|  | endline | estimate | 12.2 | -7.1 | 3.5 | -0.2 | 7.0 | 2.3 | 6.7 | -5.0 | 9.2 | -5.1 | 18.7 | -31.3 | 12.0 | -30.9 | 8.4 | -8.4 |
|  |  | 95%CI | (10.7; 13.8) | (-12.7; -1.6) | (2.5; 4.8) | (-5.7; 5.3) | (5.6; 8.7) | (-4.2; 8.7) | (6.0; 7.5) | (-8.0; -2.0) | (7.5; 11.1) | (-10.9; 0.7) | (17.6; 19.9) | (-35.2; -27.5) | (11.3; 12.9) | (-34.2; -27.6) | (6.9; 10.1) | (-14.4; -2.4) |
| Tobacco use by women |  |  |  |  |  |  | p value = 0.53 |  | p value = 0.45 | p value = 0.04 | p value = 0.34 | p value = 0.37 | p value = 0.90 | p value = 0.30 | p value < 0.001 | p value < 0.001 |  |  |
|  | baseline | estimate |  |  |  |  | 11.1 |  | 4.6 | -5.5 | 2.0 | -1.3 | 1.8 | 2.2 | 5.8 | 15.8 |  |  |
|  |  | 95%CI |  |  |  |  | (9.7; 12.6) |  | (4.0; 5.2) | (-8.3; -2.7) | (1.5; 2.7) | (-3.3; 0.8) | (1.5; 2.1) | (1.4; 3.0) | (5.3; 6.4) | (13.8; 17.9) |  |  |
|  | endline | estimate |  |  | 5.6 | 1.6 | 10.5 | -1.5 | 4.9 | -2.0 | 2.5 | -2.6 | 1.8 | 2.8 | 2.4 | 5.9 |  |  |
|  |  | 95%CI |  |  | (4.8; 6.4) | (-1.3; 4.5) | (9.4; 11.7) | (-5.3; 2.4) | (4.4; 5.4) | (-3.7; -0.3) | (1.8; 3.5) | (-4.7; -0.5) | (1.6; 2.1) | (1.9; 3.8) | (2.0; 2.8) | (4.2; 7.7) |  |  |
| Adolescent fertility rate |  |  |  |  |  |  |  |  | p value = 0.11 | p value = 0.66 | p value = 0.37 | p value = 0.14 | p value = 0.71 | p value = 0.94 | p value < 0.001 | p value = 0.02 |  |  |
|  | baseline | estimate |  |  |  |  |  |  | 89.8 | -148.2 | 77.2 | -180.2 | 99.0 | -129.6 | 71.7 | -125.5 |  |  |
|  |  | 95%CI |  |  |  |  |  |  | (81.0; 98.6) | (-194.9; -101.4) | (66.5; 88.0) | (-230.5; -129.8) | (92.2; 105.8) | (-160.5; -98.6) | (66.0; 77.5) | (-153.2; -97.8) |  |  |
|  | endline | estimate |  |  |  |  |  |  | 80.9 | -161.1 | 70.3 | -133.4 | 97.4 | -130.9 | 43.7 | -85.4 | 66.0 | -148.1 |
|  |  | 95%CI |  |  |  |  |  |  | (74.6; 87.1) | (-194.7; -127.5) | (59.7; 81.0) | (-171.0; -95.8) | (91.8; 102.9) | (-146.0; -115.8) | (41.1; 46.4) | (-103.4; -67.4) | (57.3; 74.8) | (-172.0; -124.1) |
| Under-five mortality  rate |  |  |  |  |  |  |  |  | p value = 0.52 | p value = 0.17 | p value = 0.01 | p value = 0.80 | p value < 0.001 | p value = 0.82 | p value < 0.001 | p value < 0.001 |  |  |
|  | baseline | estimate |  |  |  |  |  |  | 34.0 | -33.2 | 36.5 | -14.2 | 29.5 | -18.5 | 27.1 | -37.0 |  |  |
|  |  | 95%CI |  |  |  |  |  |  | (28.1; 39.8) | (-49.6; -16.7) | (28.1; 44.8) | (-25.8; -2.5) | (26.7; 32.3) | (-29.0; -8.0) | (23.9; 30.3) | (-44.7; -29.4) |  |  |
|  | endline | estimate |  |  |  |  |  |  | 31.7 | -21.1 | 21.9 | -10.5 | 22.7 | -20.1 | 15.4 | -13.4 | 19.9 | -4.3 |
|  |  | 95%CI |  |  |  |  |  |  | (27.9; 35.4) | (-25.5; -16.7) | (15.7; 28.0) | (-36.8; 15.8) | (20.0; 25.4) | (-28.2; -11.9) | (12.9; 17.8) | (-23.7; -3.2) | (14.6; 25.2) | (-20.0; 11.4) |
| Neonatal mortality  rate |  |  |  |  |  |  |  |  | p value = 0.85 | p value = 0.14 | p value = 0.14 | p value = 0.89 | p value = 0.01 | p value = 0.85 | p value = 0.11 | p value = 0.17 |  |  |
|  | baseline | estimate |  |  |  |  |  |  | 22.0 | -18.4 | 21.2 | -0.4 | 16.6 | -4.2 | 11.0 | -10.2 |  |  |
|  |  | 95%CI |  |  |  |  |  |  | (17.1; 27.0) | (-33.4; -3.4) | (14.6; 27.8) | (-21.2; 20.4) | (14.3; 18.8) | (-12.0; 3.6) | (9.0; 13.0) | (-14.5; -6.0) |  |  |
|  | endline | estimate |  |  |  |  |  |  | 22.6 | -5.2 | 14.8 | -2.7 | 12.7 | -5.6 | 8.7 | -1.1 | 13.1 | -3.7 |
|  |  | 95%CI |  |  |  |  |  |  | (19.2; 26.0) | (-14.3; 3.9) | (9.5; 20.1) | (-26.8; 21.5) | (10.7; 14.7) | (-16.9; 5.8) | (6.6; 10.7) | (-13.3; 11.1) | (8.6; 17.6) | (-15.3; 7.9) |
